# Comment on ‘AWGC2023 Cachexia Consensus as a Valuable Tool for Predicting Prognosis and Burden in Chinese Patients With Cancer’ by Xie et al.

**DOI:** 10.1002/jcsm.13622

**Published:** 2024-10-21

**Authors:** Xiaosong Li, Xiping Shen, Ji Wu

**Affiliations:** ^1^ Department of General Surgery Suzhou Ninth Hospital Affiliated to Soochow University Suzhou Jiangsu China

Dear Editor,

We have read a recent article titled ‘AWGC2023 cachexia consensus as a valuable tool for predicting prognosis and burden in Chinese patients with cancer’, with great interest [1]. This study is significant, as it provides insights into the association of cachexia based on the Asian Working Group for Cachexia 2023 criteria with long‐term survival. These findings have important implications for estimating survival and medical burden among Chinese patients with cancer. While recognizing the value of this study, we would like to make the following comments.

First, although the Cox regression model is widely acknowledged for its utility, it may inadvertently lead to risk overestimation with potential competing risks. Consequently, for long‐term survival prediction explored in this paper, especially when different variables are potentially interrelated, employing a competing risks model appears more fitting. Traditional survival analysis techniques might not adequately account for the influence of secondary events on the primary study endpoint, whereas the competing risks model affords a more comprehensive viewpoint [2].

Second, the authors' inclusion of important information such as demographic information and laboratory tests to adjust for potential covariates is commendable. Considering studies have shown the association of insurance status with all‐cause mortality, it can be inferred that insurance status might be an important covariate affecting the clinical outcome [3]. Additionally, factors such as nutrition condition and microvascular invasion should also be considered for a more comprehensive assessment of the stability and reliability of the results [4, 5].

Last but not least, given significant age and sex‐specific disparities in cancer incidence, a separate analysis for these subgroups could offer more nuanced insights [6].

This article is a significant step forward in our understanding of the relationship between cachexia and prognosis. A more comprehensive prediction could be an intriguing topic for further investigation. Our suggestions are merely to further refine an already outstanding piece of research.

## Ethics Statement

The authors have nothing to report.

## Conflicts of Interest

The authors declare no conflicts of interest.
